# Seaduck engineers in the Arctic Archipelago: nesting eiders deliver marine nutrients and transform the chemistry of island soils, plants, and ponds

**DOI:** 10.1007/s00442-021-04889-9

**Published:** 2021-03-06

**Authors:** N. Clyde, K. E. Hargan, M. R. Forbes, S. A. Iverson, J. M. Blais, J. P. Smol, J. K. Bump, H. G. Gilchrist

**Affiliations:** 1 Department of Biology, Carleton University, Ottawa, ON Canada; 2 Wildlife Research Division, Science and Technology Branch, Environment and Climate Change Canada, Ottawa, ON Canada; 3 Department of Biology, University of Ottawa, Ottawa, ON Canada; 4 Canadian Wildlife Service, Environment and Climate Change Canada, Gatineau, QC Canada; 5 Paleoecological Environmental Assessment and Research Laboratory, Department of Biology, Queen’s University, Kingston, ON Canada; 6 Fisheries, Wildlife, and Conservation Biology, University of Minnesota, Saint Paul, MN USA

**Keywords:** Bio-vectors, Nutrient subsidies, Stable isotopes, Arctic, Islands, Seaduck

## Abstract

Seabirds are thought to provide ecological services such as the movement of nutrients between marine and terrestrial ecosystems, which may be especially critical to productivity and diversity in nutrient-poor environments. Most Arctic ecosystems are unaffected by local human impacts and are naturally nutrient poor and especially sensitive to warming. Here, we assessed the effects of nesting common eider ducks (*Somateria mollissima*) on soil, vegetation, and pond sediments on island archipelagoes in Hudson Strait between Nunavut and Québec, Canada. Soil, moss, and pond sediments were significantly higher in nitrogen on islands with large numbers of nesting eiders compared to sites with no nesting birds. The highest concentrations of nitrogen in soils and moss occurred at the margins of ponds on eider islands, which correspond to the areas of highest eider use. δ^15^N and δ^34^S values in soils, moss, and sediments indicated substantial marine-derived organic matter inputs at the higher nutrient sites. We propose that by foraging on coastal marine benthic invertebrates and returning to islands to nest, eider ducks bio-transport and concentrate marine-derived nutrients to their colony islands, fertilizing Arctic island ecosystems in the process. As common eiders nest on thousands of low to mid-latitude islands throughout the circumpolar Arctic, these nutrient inputs likely dramatically affect biota and ecosystem functioning throughout the tundra biome.

**Supplementary Information:**

The online version contains supplementary material available at 10.1007/s00442-021-04889-9.

## Introduction

Seabirds are thought to be effective bio-vectors of nutrients from marine to terrestrial ecosystems (Anderson and Polis 1999; Mulder et al. 2011; Duda et al. 2018, 2020a, b). Through the deposition of guano, carcasses, and other detritus such as eggshells and forage items, nesting seabirds have the potential to alter the nutrient dynamics and foodwebs of terrestrial ecosystems globally (Blais et al. 2007; Wright et al. 2010; Zwolicki et al. 2016). These effects may be particularly strong in nutrient-poor environments. For example, on hyper-arid, typically nutrient-poor islands used for nesting by birds in the Gulf of California, nitrogen (N) and phosphorus (P) concentrations were up to 6 times higher in soils, while nutrient levels in some plants were up to 2.4 times greater when compared to islands without nesting seabirds (Anderson and Polis 1999).

Arctic islands generally exhibit low productivity and low food-web complexity, and therefore may respond more strongly to nutrient inputs, which would affect ecological succession, especially in areas still recovering from relatively recent glaciation. Across the Arctic, common eiders forage in the marine environment, but nest on small offshore islands to avoid terrestrial predators such as foxes (Goudie et al. 2000; Iverson et al. 2014). Thus common eiders in the circumpolar Arctic may transport marine-derived nutrients from productive coastal waters (Tremblay et al. 2012) to the severely nutrient-limited terrestrial environment of their Arctic colony islands. Such marine-derived nutrients are likely to accumulate as eiders show a high degree of philopatry to nesting sites (Goudie et al. 2000; Hargan et al. 2019). This deposition of nutrient-rich eider guano has been shown to increase nutrient concentrations in freshwaters adjacent to eider colonies (Michelutti et al. 2010; Duda et al. 2018; Hargan et al. 2019). In general, most seabird bio-vector studies have generally focused on small geographical areas with the study boundaries limited to one large seabird colony (> 10,000 nesting pairs) and the immediate adjacent aquatic and terrestrial environment (Keatley et al. 2009; Wright et al. 2010; Hargan et al. 2017). By sampling 23 islands, we investigated nutrient deposition processes that have the possibility to influence ecosystem functioning at the whole-island level across the circumpolar range of the common eider.

Seabirds occupy elevated positions in the marine food web, hence their nitrogen isotopic signatures tend to be enriched in the heavier isotope ^15^N compared to ^14^N (Anderson and Polis 1999). In addition, the lighter ^14^N isotope tends to volatilize more easily in guano than the heavier ^15^N isotope, leading to pronounced levels of ^15^N in guano (Mizutani et al. 1986). Denitrification also favours the removal of ^14^N as N_2_ or N_2_O, and ammonification can contribute to the gradual enrichment of ^15^N in the residual guano (Kendall 1998). Using nitrogen stable isotopes, studies on islands with seabird colonies have shown that seabird-derived nutrients can be found within both freshwater and terrestrial primary producers to tertiary consumers such as small rodents (Stapp et al. 1999; Caut et al. 2012; Vizzini et al. 2016). While not as commonly used as stable nitrogen isotopes, stable isotopes of sulphur can also be used as tracers for nutrient flows. In general, marine sulphur has a higher ẟ^34^S than terrigenous sulphur (Szpak et al. 2019), so ẟ^34^S may help track sources of organic matter in systems where a marine input is suspected (Peterson and Fry 1987; Hobson 1999). For example, ẟ^34^S has been used in studies to distinguish marine and terrestrial dietary sources in norway rats (Hobson 1999). However, utilizing ẟ^34^S in seabird bio-vector studies has largely been neglected to date, even though these isotopes can provide an additional useful tool in establishing transport of marine nutrient sources to terrestrial ecosystems.

We predicted that soil and plants on islands with large eider colonies would have higher δ^15^N values, δ^34^S values, and higher %N values than soil and plants from islands with few nesting eiders or reference sites completely devoid of eider nesting. If eiders are delivering marine nutrient subsidies, we further predicted that, compared to reference sites, eider-affected sites will have higher δ^15^N and %N values in the moss and soils surrounding the main freshwater ponds on each island where the eiders nest, due to fertilization from guano, feathers, and carcasses. In comparison, there is considerable overlap in δ^13^C signatures for marine primary producers and terrestrial vascular plants and mosses (Blake 1991; de la Vega et al. 2019), making stable carbon isotopes a less reliable tool in tracking the transfer of marine nutrients to land. Notwithstanding, ponds with large active eider colonies were expected to have greater %C in the surface sediments due to elevated levels of productivity caused by eider-derived nutrient inputs. Additionally, we may record ẟ^13^C enrichment in the more fertilized ponds due to increased primary productivity by algae (algae reduce their preference to incorporate ^12^C when C is in short supply). We also predicted that the isotopic signature of soil and vegetation on vegetated islands with eider colonies would more closely resemble the isotopic signature of eider guano compared to goose droppings. Goose droppings are another potential source of nutrient input on these islands, but here act more as a positive control for assessing if inputs are largely from eiders. Finally, we predicted that pond sediments on islands with large eider colonies would have significantly higher δ^15^N and %N than pond sediments on islands with few nesting eiders as not only do eiders commonly defecate on pond edges, but the ponds should act as catchments for nutrients released by eiders nesting in high densities surrounding them.

## Methods

### Study area

The islands of the Hudson Strait region present an important study opportunity as these islands are representative of thousands of similar low-lying, nearshore coastal islands across a large area of the eastern Canadian Arctic and West Greenland, many of which are used for nesting by northern common eiders (*Somateria mollisima borealis*). Whereas other studies have demonstrated seabirds that breed in very large colonies or at very high densities, and that feed at high trophic levels (e.g. are piscivorous), can be effective bio-vectors of nutrients (e.g., Keatley et al. 2011; Caut et al. 2012; Hargan et al. 2017), few studies have investigated whether seabirds that feed at lower trophic levels (e.g., feed on invertebrates) supplement nutrient levels in the terrestrial environment at or near their colonies. However, one study from the Canadian High Arctic did demonstrate that eiders deliver distinct contaminant mixtures to ponds that is distinguishable from a pond predominantly influence by nesting arctic terns (Michelutti et al. 2010). Still fewer studies have investigated whether important nutrient inputs occur across large geographical scales, such as large island archipelagos (but see Maron et al. 2006).

The islands of Hudson Strait are also unique as they have been surveyed and censused for population monitoring multiple times since the 1950s, providing us with a historical record of common eider and other wildlife use (Cooch 1986; Iverson et al. 2014). Surveys were carried out in 1956, 1976, 1980, 1981, 1983, 1991, 1997, 1998, 1999, 2000, 2002, 2004, 2009, 2010, 2011, 2012, and 2013; although, not all islands and regions were surveyed in each year. Data collected on these surveys include number of active eider nests, other species presence, and geophysical information such as island size, distance from shore, and elevation.

### Site selection

Using records from the historical eider surveys, we selected candidate islands prior to conducting our work to attempt to minimize variation in their physical criteria as much as possible (e.g. distance from coastal shoreline, area, elevation—see Appendix 1), although this was not always possible. For instance, it was challenging to find large islands that had few nesting common eiders, or small islands with large colonies. We were also not always able to visit all candidate islands due to logistical and environmental conditions, such as local sea ice or inclement weather. We also attempted to maximize the spatial extent of our sampling as much as possible within the logistical constraints of our sampling method.

Islands were then divided into two treatment groups based on recent eider use (see Table 1). Islands with large numbers of recent active eider nests and clear evidence of an eider colony were included in the ‘high eider’ group (*n* = 17), whereas islands with very few nests and no evidence of a colony were placed in the ‘low eider’ group (*n* = 5). We also included a third group in our analyses, which we called ‘reference’ (*n* = 14, but sample size varies across moss, soil, and sediments, see Table 1). These eider-free sites were located on the nearest large terrestrial landmass adjacent to the islands that we chose in the two groups above. Reference sites were chosen to provide a control group where we expected the nutrient sources to be of terrestrial origin, but that were also nearby to our study locations. These sites were located along the shorelines of each region where islands were being sampled and were taken from a similar distance from shore as island samples. We treated Digges Island sites as reference sites because of the island’s large area (~ 92 km^2^) and lack of eider nesting activity.

**Table 1:** List of islands and reference sites sampled for this study

ID code	Region	# Surveys	Active nests (most recent) + (year)	Active nests (average)	SE (average nests)
High eider					
A-044	Baffin Island	7	141 (2012)	81	26
A-045	Baffin Island	7	15 (2012)	164	100
A-054	Baffin Island	5	243 (2012)	93	40
A-056	Baffin Island	7	434 (2012)	263	74
A-083	Baffin Island	2	259 (2010)	162	97
A-085	Baffin Island	1	225 (2010)	225	N/A
A-108	Baffin Island	2	234 (2010)	125	110
A-110^a^	Baffin Island	2	91 (2010)	150	59
A-112^a^	Baffin Island	2	251 (2010)	139	113
A-114	Baffin Island	1	197 (2010)	197	N/A
A-135	Baffin Island	6	127 (2012)	465	89
A-136	Baffin Island	3	444 (2011)	268	88
D-003	Digges Sound	1	228 (2012)	228	N/A
D-004	Digges Sound	1	212 (2012)	212	N/A
D-012	Digges Sound	5	230 (2012)	147	56
D-016	Digges Sound	2	367 (2012)	197	171
D-022	Digges Sound	1	101 (2012)	101	N/A
Low eider					
A-038	Baffin Island	1	7 (2011)	7	N/A
A-043^b^	Baffin Island	5	3 (2012)	10	7
A-143^b^	Baffin Island	2	6 (2011)	6	1
D-007^c^	Digges Sound	1	8 (2012)	8	N/A
D-019	Digges Sound	2	11 (2012)	17	6
Reference					
Cape Dorset (outside town)^b^	Baffin Island	N/A	0 (N/A)	0	N/A
Ivujivik (outside town)	Digges Sound	N/A	0 (N/A)	0	N/A
Baffin reference #1^b^	Baffin Island	N/A	0 (N/A)	0	N/A
Baffin reference #2^b^	Baffin Island	N/A	0 (N/A)	0	N/A
Baffin reference #3^b^	Baffin Island	N/A	0 (N/A)	0	N/A
DI-1^c^	Digges Sound	N/A	0 (N/A)	0	N/A
DI-2^c^	Digges Sound	N/A	0 (N/A)	0	N/A
DI-3^c^	Digges Sound	N/A	0 (N/A)	0	N/A
DI-4^c^	Digges Sound	N/A	0 (N/A)	0	N/A
DI-5^c^	Digges Sound	N/A	0 (N/A)	0	N/A
DI-6^c^	Digges Sound	N/A	0 (N/A)	0	N/A
DI-7^c^	Digges Sound	N/A	0 (N/A)	0	N/A
DI-8^c^	Digges Sound	N/A	0 (N/A)	0	N/A
DI-9^c^	Digges Sound	N/A	0 (N/A)	0	N/A

Islands and reference sites were placed into groups based on the amount of common eider nesting activity. High Eider sites were islands with large, obviously utilized common eider colonies present. Low Eider sites were islands that had little evidence of eider nesting activity. Reference sites were included to provide comparisons for alternative sources of nutrients. Data on nesting activity were taken from historical surveys of the region from the period 1956–2012

^a^Pond(s) present, but sediments not collected

^b^No pond present at this site. No sediments collected or analysed

^c^Only pond sediments collected at this site, no soil or moss collected or analysed

We grouped sites in the previously described manner as we were primarily interested in the difference between islands with a large eider influence (high eider) and islands without (low eider) but wanted a third group that was independent of any eider influence for comparison (reference). Ideally we would have sampled from islands with no eider nests for our ‘low eider’ group, but to do so would have required sampling islands too small to be comparable to ‘high eider’ islands as any island with zero nests on it was usually too small or low in elevation that it would have been awash at high tide or during winter storms. Despite this, the ‘low eider’ islands were clearly different than islands from the ‘high eider’ group as the ‘high eider’ islands had large, established colonies where the density of nest sites was extremely high, while the ‘low eider’ islands had no evidence of a colony and a few lone nests scattered across the island. We avoided sampling islands that historically had large colonies that have disappeared in modern times, as these islands would have been difficult to place into either of our treatment groups.

### Field sampling

**Fig. 1:**
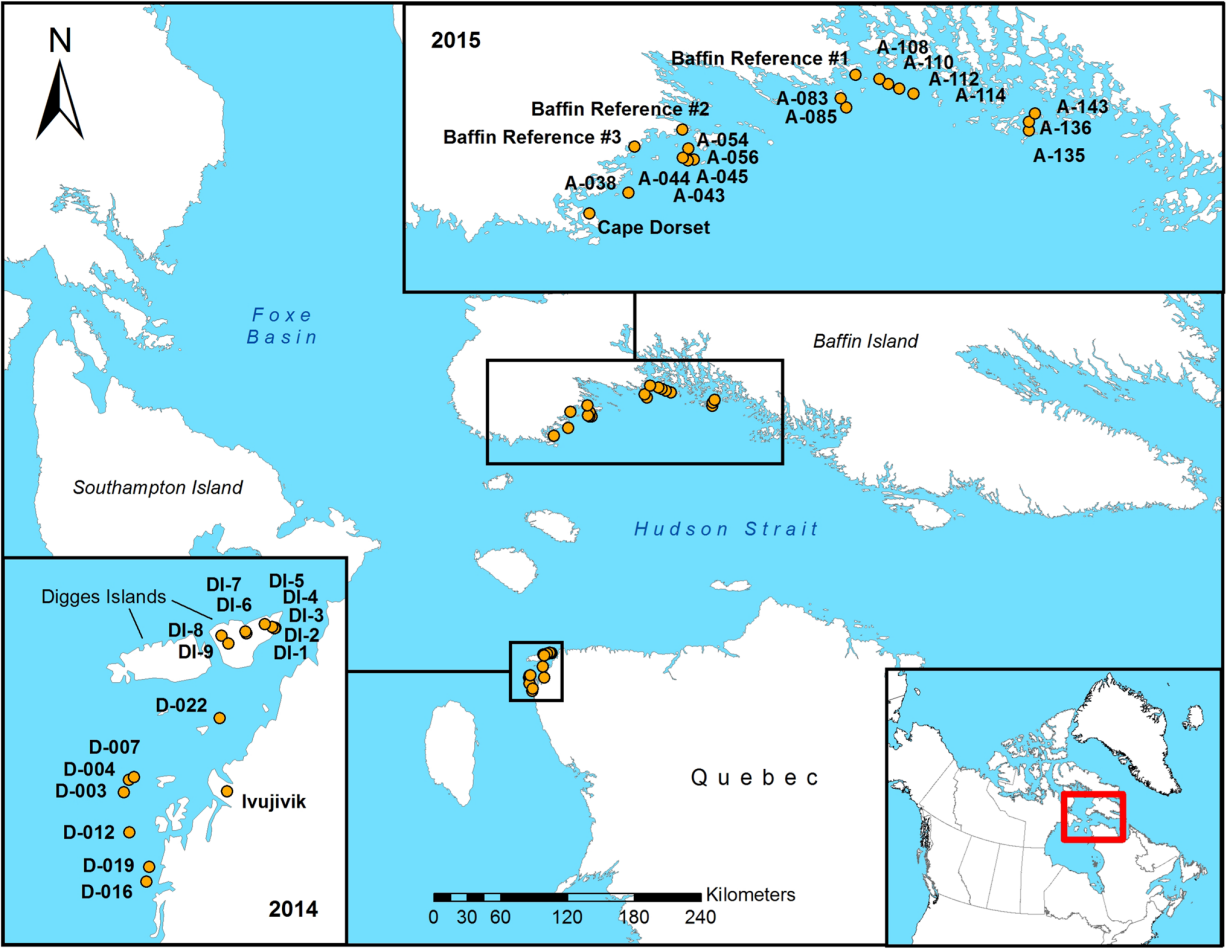
Study site and sampling locations. The field work for this project took place in 2013, 2014, 2015 and 2016. Surveys were completed by boat with the assistance of local guides from Cape Dorset, Nunavut and Ivujivik, Quebec. Samples were also collected from the East Bay island migratory bird sanctuary and from lakes and ponds on Digges Island

Fieldwork took place during the summer of 2013, 2014, 2015 and 2016. In 2013, the sediments of one lake in the community of Ivujivik and nine lakes on the northern side of Digges Island East were sampled by helicopter (sites with DI prefix—see Fig. 1). For this study, these lakes have been grouped with the reference sites since Digges Island is substantially larger than the study islands and has no nesting eiders present. In 2014, 2015 and 2016, coastal islands in Digges Sound, Québec (sites with D prefix—see Fig. 1) and east of Cape Dorset, Baffin Island (sites with A prefix—see Fig. 1) were accessed by boat with the aid of local Inuit guides.

On our island surveys, a team of 3 to 5 people landed by boat at each study island to complete bio-physical surveys. On islands with colonies, we sampled the main pond that typically occurs at the centre of the colony on each island. We identified this pond by surveying the entire island and then selecting the pond surrounded by the highest density of eider nests. Most islands only had one or two ponds on them, so it was usually quite easy to determine which pond to sample. To standardize choice of ponds on islands with very little or no nesting activity, the largest pond furthest from the ocean was selected for sampling. Once a selection was made, a 10 cm × 10 cm sample of moss and soil was excavated using a trowel from a random location selected ad hoc within 2 m of the main pond for isotope analysis. On islands with large eider colonies the main pond was always surrounded by a large fringe of vegetation, which provided a large area from which to sample. On islands with few eider nests, this was not usually the case, and we were often forced to sample whatever vegetation we could find within 2 m of the pond. Coastal reference sites were sampled opportunistically during the field seasons where safe landings could be made, though effort was made to ensure they were spread out over the study area as best as possible (see Fig. 1). Samples were stored in sealed plastic bags and frozen upon returning from the field, until analysis. Pond sediments were collected using a sediment push corer and the cores were sectioned into 0.5 cm intervals at site (Glew 1988; Glew and Smol 2016). Sediment for stable isotope analysis was taken from the surface samples (0–0.5 cm) of each core.

Goose droppings were commonly observed on islands with large eider colonies and were collected opportunistically from several islands for isotopic comparison to eider guano to rule out goose droppings as a major alternative source of nutrients. No other animals or their droppings (e.g. gulls, terns, bears) were observed in any large numbers on islands with large eider colonies. Eider droppings were collected in 2015 at the long-term research site at the East Bay Migratory Bird sanctuary, as they do not persist on the landscape as readily as goose droppings.

Additional moss and soil collections were also completed on a subset of 5 ‘high eider’ islands on the coast of Baffin Island (A-045, A-048, A-056, A-101, and A-135). These collections were completed in the summer of 2016, other than A-135, which was collected during the 2015 field season. These islands were selected to represent a sample of large eider colony islands across the geographic range of the survey area in the Baffin Island region. On each island, 30 m transects were laid out in each of the 4 cardinal directions beginning at the edge of the main pond. Samples of moss and soil were collected every 5 m along the transects to examine the relationship between stable nitrogen isotope values and nutrient levels in relation to patterns of eider habitat use across the islands. Samples of soil and moss were not always available within each quadrat; however, in general, all islands and distances were represented (sample breakdown by distance—0 m: *n* = 10, 5 m: *n* = 7, 10 m: *n* = 6, 15 m: *n* = 7, 20 m: *n* = 7, 25 m: *n* = 7, 30 m: *n* = 6).

### Stable isotope analysis

All vegetation samples were manually cleaned of debris and other particles, freeze dried and homogenized using a ball mill or mortar and pestle before being analysed for stable isotopes. After homogenization, samples and standards were weighed into tin capsules and loaded into an elemental analyser (Isotope Cube, Elementar, Germany) interfaced to an isotope ratio mass spectrometer (IRMS) (Delta Advantage, Thermo, Germany) then flash combusted at 1800 °C (Dumas combustion). The resulting gas products were carried by helium through columns of oxidizing/reducing chemicals optimised for CO_2_ and N_2_. The gases were then separated by a purge and trap adsorption column and sent to the IRMS interface (Conflo III, Thermo, Germany) then to the IRMS. All analyses were performed at the Ján Veizer Stable Isotope Laboratory (formerly G. G. Hatch) at the University of Ottawa, Canada. The standards used in the analysis were atmospheric nitrogen (δ^15^N), Cañon Diablo meteorite (δ^34^S) and Pee Dee Belamnite limestone (δ^13^C).

Standard isotope values were calculated using the formula:

$$\delta = \left( {R_{{{\text{sample}}}} /\left( {R_{{{\text{standard}}}} - 1} \right)} \right) \times 1000,$$

where *R* = the ratio of ^15^N/^14^N, ^34^S/^32^S or^13^C/^12^C.

### Statistical analysis

Data on δ^15^N, δ^34^S, δ^13^C, %N, %S and %C in both moss and soil were compared between the three treatment groups (high eider, low eider and reference) using one-way ANOVAs and Tukey post-hoc tests to test for differences between groups. Because of unequal sample sizes between treatment groups, we used Levene’s test of equality of variances to confirm our data were suitable for these analyses. Data on δ^15^N, δ^13^C, %N and %C in pond sediments were compared between the same three treatment groups, with the addition of Digges Island sites to the reference category (DI sites—see Table 1).

Linear regression was used to investigate trends in isotope signatures and nutrient concentrations relative to the number of active nests on islands. In these analyses, the most recent number of active nests from previous surveys was used as the independent variable, with isotope values or % nutrient values as the response variables. Reference sites were excluded from this analysis as we were interested in determining if the amount of nutrient deposition was proportional to the number of birds using an island. To investigate the within-island trends in isotope signatures and nutrient levels relative to distance from areas of high eider use we performed linear regressions. In this analysis, distance to pond edge was used as the independent variable, with isotope ratios or % nutrient values as the response variable. To compensate for the lack of samples from some quadrats, data for the same distance from ponds were pooled across islands. Sampling site was included as a variable in our models, and data were only pooled from islands where no significant effect of sampling site was detected. All statistical tests were performed in R (© 2015 The R Foundation for Statistical Computing).

## Results

### Comparisons across locations

**Fig. 2:**
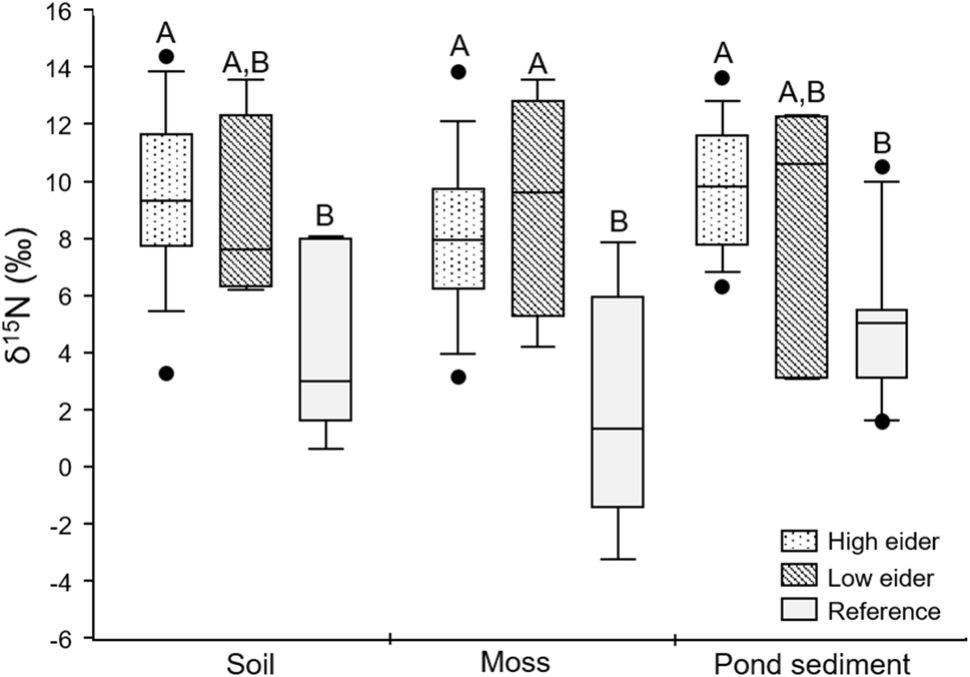
Differences in δ^15^N values (‰) in soil, moss and pond sediments across the three treatment groups. Treatment group had a significant effect on δ^15^N values in both soil, moss and pond sediments. Letters A and B indicate differences between groups: those that share a letter are not significantly different from one another

We investigated δ^15^N values in soil, moss, and pond sediments as a tracer of high trophic nutrients to islands. As predicted, treatment group was a significant predictor of δ^15^N values in soil (*F*_22_ = 5.528, *p* = 0.008, Fig. 2). There were significantly higher δ^15^N values (+ 5.3‰) in soil on islands with large eider colonies compared to reference sites (Tukey Post-hoc: *p* = 0.008), with ‘low eider’ islands not significantly different from either ‘high eider’ or reference sites. Treatment group was a significant predictor of δ^15^N values in moss (*F*_24_ = 7.764, *p* = 0.003, Fig. 2). On both ‘high eider’ and ‘low eider’ islands there were significantly higher δ^15^N values in moss samples (+ 6.2 and + 7.1‰, respectively) compared to reference sites (Tukey Post-hoc: *p* = 0.003 and 0.01, respectively). Treatment group was also a significant predictor of δ^15^N values in pond sediments (*F*_25_ = 11.76, *p* < 0.001, Fig. 2). Pond sediments on ‘high eider’ islands were significantly enriched (+ 5.1‰,) compared to reference sites (Tukey Post-hoc: *p* < 0.001). ‘Low eider’ sites were not significantly different from either ‘high eider’ or reference sites.

**Fig. 3:**
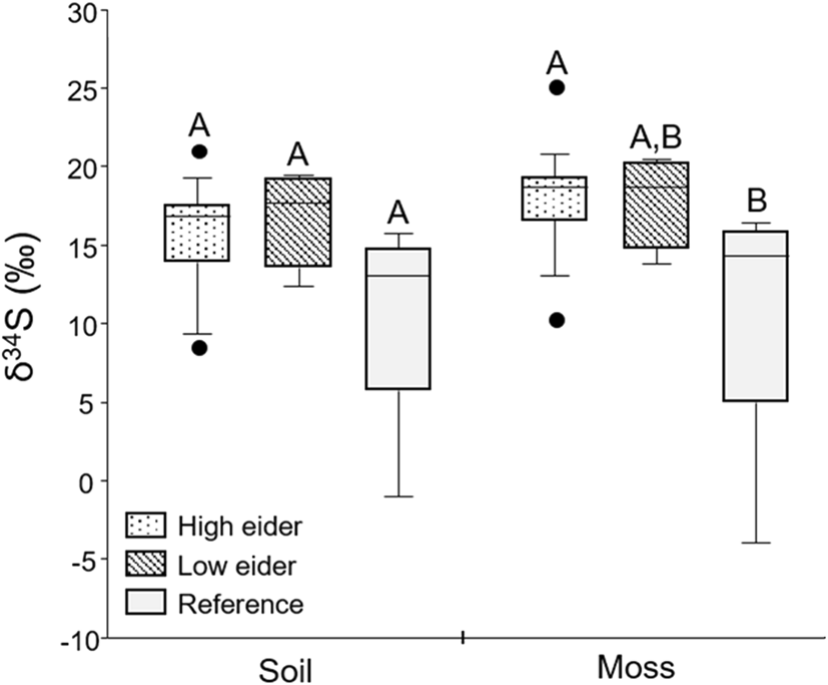
Differences in δ^34^S values (‰) in soil and moss across the three treatment groups (it was not possible to obtain δ^34^S values for pond sediments due to sample quantity restrictions). Treatment group had a significant effect on δ^34^S values in moss, but not soil. Letters A and B indicate differences between groups: those that share a letter are not significantly different from one another

We investigated δ^34^S values in soil and moss as a tracer of marine nutrient input to islands. Contrary to our predictions, treatment group was not a significant predictor of δ^34^S values in soil (Fig. 3) but was in moss (*F*_24_ = 4.759, *p* = 0.02, Fig. 3). There were significantly higher δ^34^S values (+ 6.7‰) in moss samples on ‘high eider’ islands compared to reference sites (Tukey Post-hoc: *p* = 0.02), but no significant difference between ‘low eider’ islands and reference sites.

We also investigated δ^13^C values in soil, moss, and pond sediments as a third discriminatory factor of nutrient input to islands. As predicted, treatment group was not a significant predictor of δ^13^C values in soil or moss, but it was a significant predictor of δ^13^C values in pond sediments (*F*_25_ = 6.73, *p* = 0.005). δ^13^C values were significantly higher in pond sediments on ‘low eider’ islands (although sample size was small, *n* = 3) when compared to ‘high eider’ islands (+ 6.7‰) and reference sites (+ 7.7‰) (Tukey Post-hoc: *p* = 0.008 and 0.004, respectively).

**Fig. 4:**
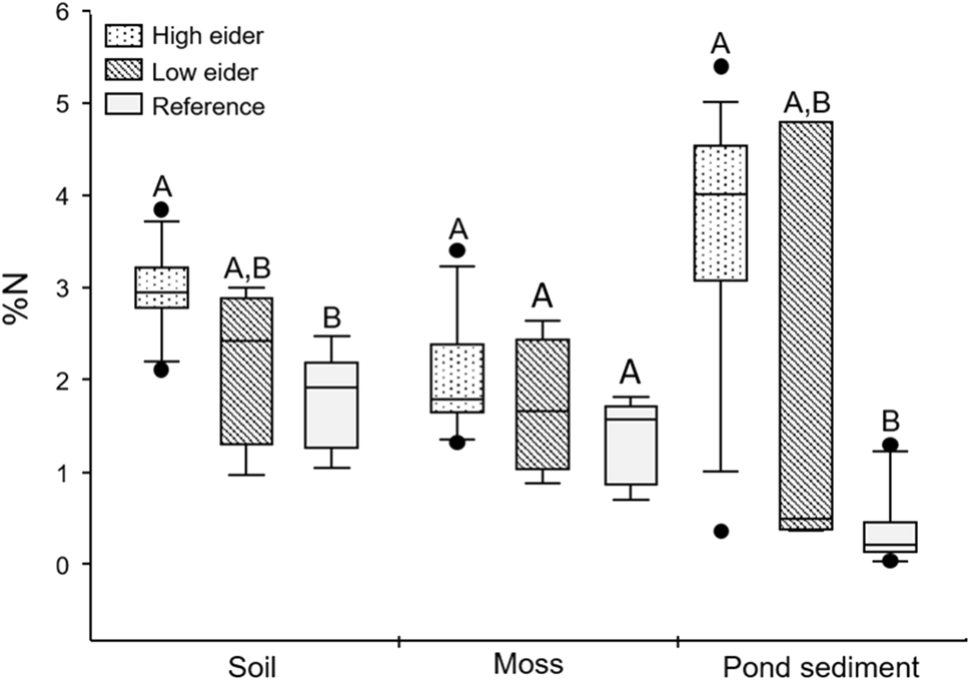
Differences in percent nitrogen (%N) values in soil, moss and pond sediments across the three treatment groups. Treatment group had a significant effect on %N values in both soil, moss and pond sediments. Letters A and B indicate differences between groups: those that share a letter are not significantly different from one another

Percent nitrogen was measured in soil, moss and pond sediments as an indicator of the amount of nitrogen transported from the marine environment to colony islands. Treatment group was a significant predictor of %N values in soil (*F*_22_ = 4.704, *p* = 0.02, Fig. 4) and marginally non-significant in moss (*F*_24_ = 2.994, *p* = 0.07, Fig. 4). There were significantly higher %N values in soil collected from ‘high eider’ islands with large eider colonies compared to reference sites (Tukey Post-hoc: *p* = 0.02), with ‘low eider’ islands intermediate between the two. There were marginally non-significant differences in %N values in moss on ‘high eider’ islands with large colonies compared to reference sites (Tukey Post-hoc: *p* = 0.07). Compared to reference sites, moss samples on ‘high eider’ islands with large colonies had ~1.5 times more nitrogen (2.05 vs 1.34%) and soil samples had ~1.5 times more nitrogen (2.80 vs 1.76%). Treatment group was also a significant predictor of %N values in pond sediments (*F*_25_ = 19.52, *p* < 0.001). We observed significantly higher %N values in pond sediments on ‘high eider’ islands when compared to reference sites (Tukey Post-hoc: *p* < 0.001). Compared to reference sites, pond sediments from ‘high eider’ islands had ~10 times more nitrogen (3.58 vs 0.35%).

Percent sulphur was measured in soil and moss samples as a potential indicator of marine nutrient input. There was no significant trend in %S values in soil or moss across the three treatment groups.

Percent carbon was measured in soil, moss, and pond sediments mainly as an indicator of productivity in ponds on islands. We observed no significant difference in %C values in soil or moss between ‘high eider’ or ‘low eider’ islands or reference sites, however treatment group was a significant predictor of %C values in pond sediments (*F*_25_ = 23.70, *p* < 0.001). There were significantly higher %C values in pond sediments on ‘high eider’ islands when compared to reference sites and ‘low eider’ sites (Tukey Post-hoc: *p* < 0.001 and *p* = 0.02, respectively). Pond sediments on ‘high eider’ islands had ~8 times higher carbon concentrations than reference sites (34.73 vs 4.52%) and ~2 times more carbon than nearby ‘low-eider’ islands (34.73 vs 15.09%).

**Fig. 5:**
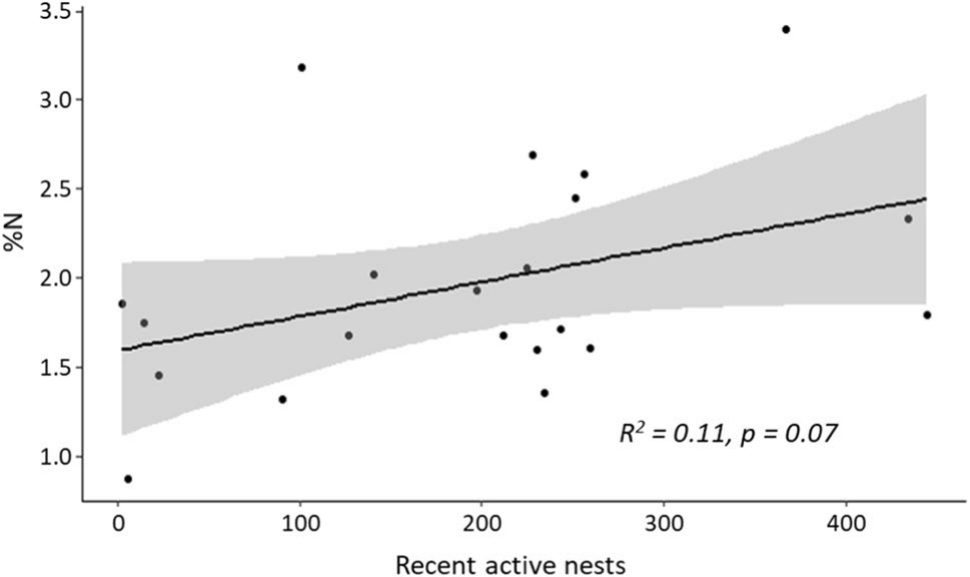
The trend in percent nitrogen (%N) in relation to number of recent active common eider nests across all islands. This trend was marginally not significant; however, a general trend of increasing %N with increasing recent active nests is evident

To assess whether the amount of nutrient input to islands increased in relation to increasing number of nesting eider ducks, we compared δ^15^N and %N levels across all island sites. %N in moss tended to increase with the number of recent active nests (Fig. 5), but the slope was marginally not different from 0 (*n* = 20, *p* = 0.07, *R*^2^ = 0.11). We observed no evidence of a trend between number of recent active eiders and soil %N across all sites. There was also no trend in δ^15^N, %C or %S in moss or soil across all sites relative to the number of active eider nests.

### Within-island comparisons

**Fig. 6:**
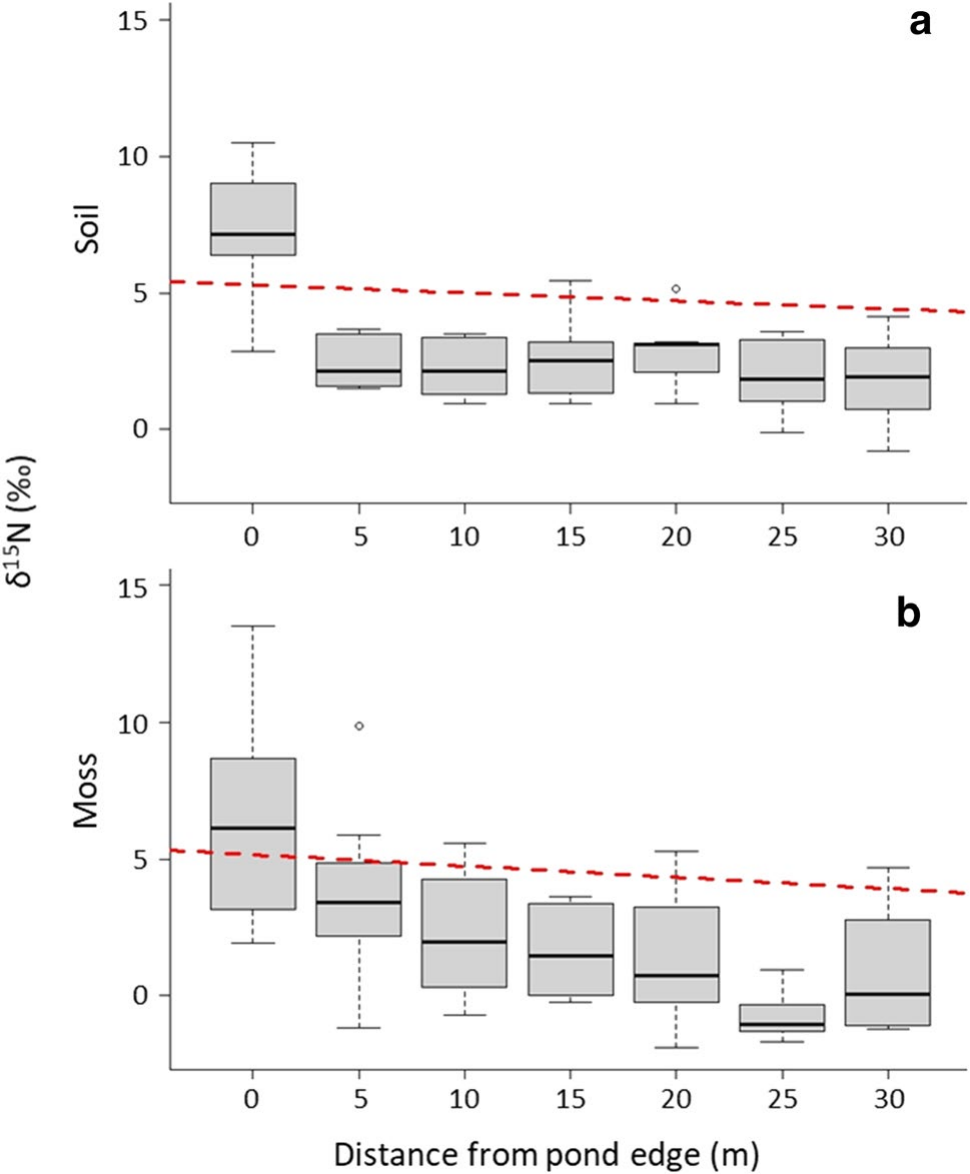
The within-island trend in δ^15^N (‰) in **a** soil and **b** moss in relation to distance from the main pond on a subset of islands. There was a significant decreasing trend in δ^15^N in both soil and moss samples as distance from the edge of the main pond increased, suggesting a source of high trophic-level nutrients at the area corresponding to high eider use

**Fig. 7:**
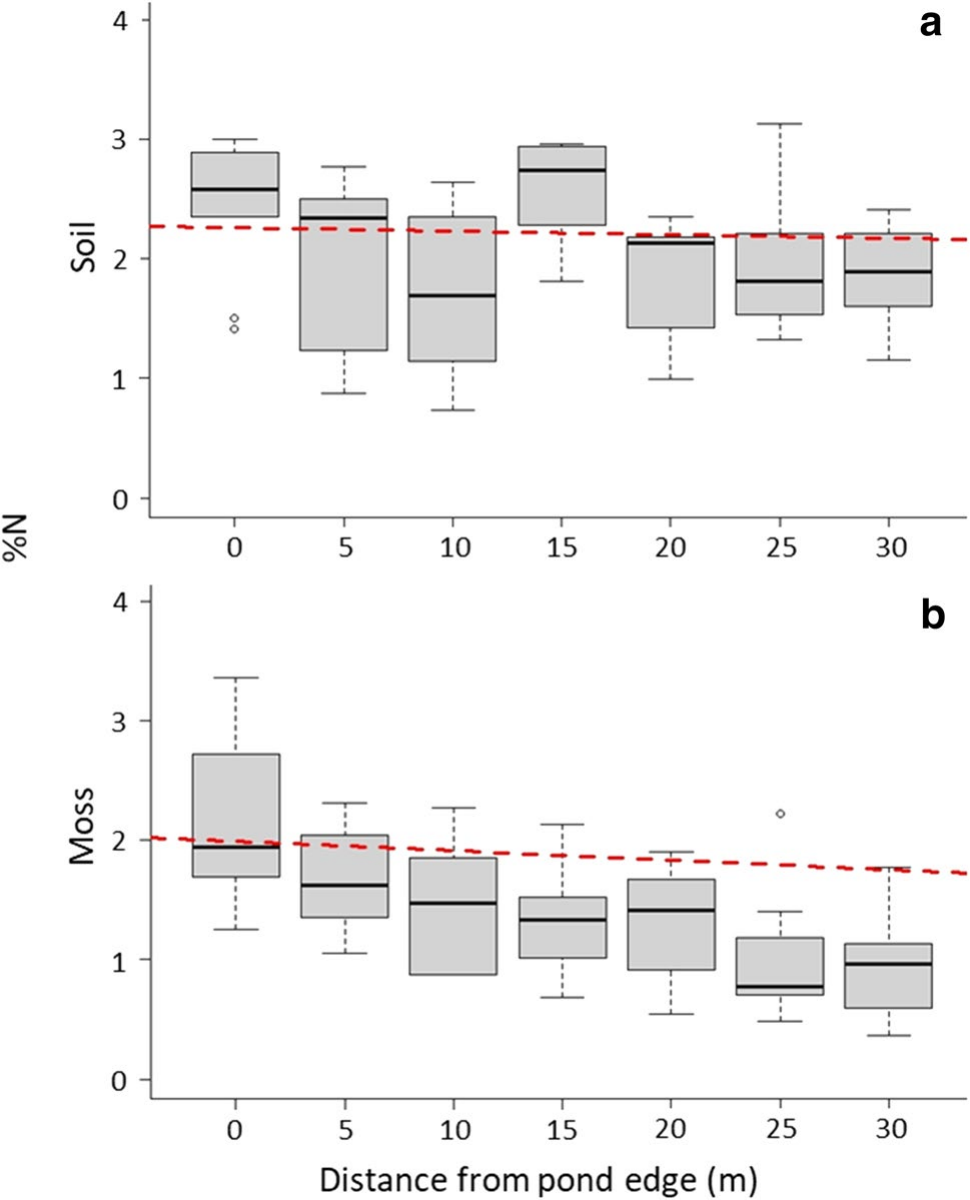
The within-island trend in percent nitrogen (%N) values in **a** soil and **b** moss in relation to distance from the main pond from a subset of islands. There was a significant decreasing trend in %N levels in moss samples as distance from the edge of the main pond increased, suggesting higher rates of nutrient deposition at the area corresponding to high eider duck use. This trend was not observed in soil samples

We investigated the within-island relationships of δ^15^N and %N relative to areas of high eider use on 5 ‘high eider’ islands (*n* = 50 samples) to determine if patterns of nutrient inputs were related to the patterns of eider duck habitat use. As predicted, δ^15^N values of both soil and moss were greatest around pond margins (Fig. 6) and decreased significantly moving away from the main pond area of high eider use (*p* < 0.001, *R*^2^ = 0.36 and *p* < 0.001, *R*^2^ = 0.36, respectively). We observed no significant effect of distance from pond edge in soil %N levels (*p* = 0.09, Fig. 7), though %N values for moss were significantly elevated at pond margins (*p* < 0.001, *R*^2^ = 0.35). We were unable to investigate the effect of distance from pond edges on islands with no eider nesting activity as there was very little soil or vegetation to sample on those islands.

### Dropping analyses

We compared the δ^15^N of goose droppings to eider duck droppings to determine if goose droppings were a confounding factor in our study system. Common eider droppings were significantly enriched in δ^15^N values compared to goose droppings (+ 6.18‰, *n* = 11, *df* = 4.11, *p* = 0.002). Thus, common eider droppings had similar δ^15^N values to the soil and moss samples collected on colony islands (δ^15^N eider droppings = 10.09‰, moss = 8.29‰, soil = 9.71‰). Goose droppings were significantly enriched in δ^34^S compared to eider droppings (+ 3.83‰, *n* = 11, *df* = 4.10, *p* = 0.02); however, values for eider droppings were closer to ecosystem components on colony islands than goose droppings (δ^34^S eider droppings = 16.54‰, goose droppings = 20.37‰, moss = 17.93‰, soil = 15.17‰).

## Discussion

### Comparisons across locations

The results of our multi-isotope approach and elemental analyses point to the presence of isotopically enriched nutrient pools within the terrestrial and aquatic environments on islands supporting large common eider nesting colonies. δ^15^N values in soil, vegetation, and sediment samples from ‘high eider’ islands with large colonies were significantly enriched compared to reference sites (Fig. 2), indicating nutrient input from a higher trophic level. Seabirds forage at high trophic levels and have been shown to be responsible for increases in δ^15^N values in other systems (Anderson and Polis 1999; Caut et al. 2012; Gonzalez-Bergonzoni et al. 2017; Mosbech et al. 2018). Common eiders forage on benthic invertebrates (Goudie et al. 2000) and thus could be responsible for the input of trophically enriched nitrogen onto islands where they nest. These results align well with the hypothesis that eiders transport nutrients to these Arctic islands and in doing so engineer important ecological changes that accelerate soil development and succession by primary producers.

An interesting and unexpected finding was that δ^15^N values in moss on ‘low eider’ islands were not significantly lower than δ^15^N values in moss on ‘high eider’ islands. One explanation for this result is the possibility that over time even a very small number of eider ducks are capable of transporting enough nutrients to these islands to be detected by our analysis. This is a reasonable possibility, as ‘low eider’ islands in our study had a small number of birds nesting on them (see Table 1). This is because islands that were large enough for reasonable comparison to islands with large eider colonies always had a few birds nesting sporadically on the islands. Any island small enough to have no eider nesting activity would not have been suitable for comparison to the larger islands with eider colonies. Further to this point, we had to actively search for vegetation to sample on ‘low eider’ islands, so it is possible we were unintentionally biasing our sample collection to localized areas on those islands that had some level of nutrient availability. We interpret these findings to mean that even on islands lacking large active colonies, there can still be pockets of nutrient enrichment historically deposited by the few eider ducks nesting there. This trend was not observed for δ^15^N values in soil, potentially because moss carpets in Arctic tundra have been shown to absorb nutrients before they can infiltrate the soil below and we were only sampling surface soils (Pouliot et al. 2009). δ^15^N appeared to be unaffected by the number of nests on islands, which is intuitive, as all eiders are feeding at the same trophic level, so islands with more individuals will not necessarily increase the δ^15^N signature present once it is established and detectable.

There were significantly higher δ^34^S values in moss on ‘high eider’ islands compared to reference sites, with ‘low eider’ islands falling somewhere in the middle (Fig. 4). This increase in δ^34^S values indicates a marine source of nutrients that were not present at reference sites. Reference sample sites were located as close, or closer to the ocean than most samples sites on islands, so sea-spray would have been similar between sites and is likely not the explanation for these results. Previous work has shown that plants grown in greenhouses that are fertilized with seabird guano have enriched δ^34^S values when compared with control plants (Szpak et al. 2019). These results again align well with the hypothesis that eiders transport marine nutrients to these islands as eiders forage primarily on marine benthic invertebrates which are enriched in the heavier isotope of sulphur as they obtain their nutrients from the marine environment (Hobson 1999).

Percent nitrogen in soil and sediment samples were significantly higher on ‘high eider’ islands compared to reference sites, with ‘low eider’ islands again found to be intermediate between the two (Fig. 4). While there was no significant difference in percent nitrogen in moss samples, there was strong evidence for a trend. Soil samples from ‘high eider’ islands were on average 52% enriched in nitrogen as compared to reference sites while moss samples were enriched by 60%. For example, on one ‘high eider’ island percent nitrogen levels in moss samples were ~5 times higher compared to the lowest reference site. Other studies investigating seabird nutrient transport have found remarkably similar results. Anderson and Polis (1999) found 55–145% nitrogen enrichment on desert islands in the Gulf of California, USA while Smith (1978) found plants influenced by seabirds were 55% more enriched in nitrogen on sub-Antarctic Marion Island, South Africa. These results support our hypothesis that eider ducks are transporting marine-derived nitrogen to islands where they nest. This increase in percent nitrogen levels in soil and moss is likely a function of scale; only a few birds are needed to modify the δ^15^N signature (as recorded in our samples from non-colony islands with several nesting birds) but the *percent* nutrient levels likely increase according to the number of active nests (see Fig. 5). Though the relationship we found between number of nests and percent nitrogen levels was marginally non-significant, we argue that it is intuitive that the amount of nitrogen transported to islands via by eider ducks would increase as the number of nesting birds increases—even if our methods were not sensitive enough to detect this increase. Taken together, these results lead us to conclude that only a small number of nesting eiders can alter the isotopic makeup of the communities of the islands they nest on, but a larger number of active nests is required to have a measurable impact on the overall nutrient levels on these islands.

### Within island comparisons

We observed evidence of spatial variation in nutrient deposition, particularly related to the proximity of ponds. δ^15^N values were highest in both soil and moss samples collected nearest the main pond on each island, and both declined with distance from the pond margins (Fig. 5). This observation supports our hypothesis that eiders transport nitrogen to colony islands through their excrement, as the main pond and the surrounding areas receive the highest amount of eider guano due to the concentration of nesting birds as they arrive and depart the island (Fast 2006). Gravid females use these ponds as landing zones as they have very-high wing loading and risk injury when landing on solid ground at this time of year. Females also tend to spend some time on these ponds with ducklings after hatching, though broods do depart the ponds for the safety of the ocean within a few hours or days. Males paired with females will also use the pond as a landing site as they closely follow their mate, whereas single males and failed breeders congregate around the pond margins to loaf and sleep en masse. Because of this, the main pond of each island is almost always occupied by multiple females and their mates, as well as unpaired males seeking mates, failed breeders, and eventually duckling and attendant hens. Most birds defecate within and upon exiting the pond, leading to a large influx of fresh nutrient-rich droppings to this area each summer. Metals and nutrients were elevated in ponds on islands with large eider colonies compared to non-colony islands in this study system (Duda et al. 2018). In addition, the many birds utilizing the areas immediately surrounding the pond margins as loafing sites during the pre-breeding and breeding season deposit droppings, further adding to the deposition of nutrients in this area over time. Because of these behavioural characteristics of eider ducks, the areas surrounding these ponds are likely to receive the highest amount of guano deposition. We interpret these findings as further evidence to suggest that eider ducks are the source of these increased nutrient levels.

Alternative explanations of higher concentrations of nutrients near the pond edge include runoff from other parts of the island, but we argue this is unlikely the main driver of this pattern for two reasons. One, these islands are relatively low-lying, with limited topography to generate much runoff into the ponds. Some of the ponds we surveyed were located at the very top of islands, so any nutrients would be washed away, rather than toward the pond; and two, while there may be some local movement of nutrients towards the ponds within their small catchment areas, we suggest that the source of these nutrients would likely still be eider-related as these catchment areas are heavily utilized for nesting and loafing as mentioned previously. This hypothesis is supported by our experience while sampling these islands, as we were regularly unable to find vegetation or soil to sample on islands without large numbers of nesting eiders. This provides further evidence that eider-transported nutrients are fundamental to the functioning of these island ecosystems.

### Dropping analyses

The results of the dropping analysis also indicate that herbaceous geese, the only other abundant animal in this system, are unlikely to be the bio-vector responsible for these increases in nutrient levels. δ^15^N values measured in common eider droppings were more similar to those found in soil and moss on colony islands (δ^15^N droppings = 10.09‰, moss = 8.29‰, soil = 9.71‰), and eider droppings were significantly more enriched in δ^15^N than goose droppings (+ 6.18‰). In subarctic ponds impacted solely by nesting geese, the sediment δ^15^N values were < 1.0‰, demonstrating that the terrestrial herbaceous diet of geese yields little enrichment in δ^15^N values (MacDonald et al. 2015). As such, we believe goose droppings would be unable to result in the changes in δ^15^N values we observed in moss and soil. Our findings for δ^34^S are less clear, as eider droppings had values more similar to moss and soil, but goose droppings were surprisingly enriched more than eider droppings (δ^34^S eider droppings = 16.54‰, goose droppings = 20.37‰, moss = 17.93‰, soil = 15.17‰). Geese are also an unlikely source of nutrients on these islands as they are generally only transient visitors to these islands while en route to breeding colonies further north, and thus do not spend large amounts of time on the islands compared to nesting eiders over an entire breeding season. We did not observe evidence of other animals (e.g. gulls, terns, bears) or their droppings in large numbers on any island with large eider colonies, and thus chose not to include them in our list of potential nutrient sources.

Seabirds have been shown to be effective bio-vectors of nutrients from marine sources to colony sites on islands in several studies worldwide (Michelutti et al. 2009; Keatley et al. 2011; Caut et al. 2012; Zmudczyńska-Skarbek et al. 2015; Mosbech et al. 2018). Our results are a clear indication that common eider ducks transport high trophic level marine-derived nutrients from productive ocean waters to the terrestrial environment of the islands where they nest. The elevated δ^15^N and δ^34^S signatures and %N values that we detected are strong evidence that nutrient inputs from marine environments to these island systems occurs via a bio-vector that feeds at mid-trophic levels. The absence of these isotopic signatures on nearby reference sites without nesting eiders indicates that a unique process is transporting these nutrients to these island sites. Finally, the spatial patterns of intra-island isotope and %N responses closely align with the habitat use patterns of eider ducks.

Our results, taken together, strongly suggest that common eider ducks are acting as ecologically important bio-vectors of marine nutrients to the terrestrial environment of the Arctic islands where they nest, both in small numbers and large. As common eider ducks nest throughout Hudson Strait, including Ungava Bay and the surrounding areas (Cooch 1986; Iverson et al. 2014), our study demonstrates that these marine-derived nutrients could have broad regional-scale effects on soil and water chemistry across these island archipelagos.

Due to the severely nutrient limited nature of terrestrial and aquatic Arctic tundra communities (Fox 1992; Shaver and Chapin 1995), even minor increases in soil and aquatic nutrient levels have the potential to have dramatic effects on primary producers and therefore the entire ecosystem through indirect effects. These large-scale effects on ecosystem functioning suggest that common eiders act as an ecosystem engineer by enriching the nutrient levels of the aquatic and terrestrial ecosystems through the deposition of large amounts of droppings on islands where they nest in numbers. Additionally, eider nutrient inputs may be of particular importance to the formation of the ecosystems on islands in this region because the low-lying island archipelagos of northern Hudson Bay and Hudson Strait were likely covered by the Laurentide ice sheet until at least ~ 10,000 years ago.

Nutrient transport via common eider droppings may have played an integral role in the development of the current-day productive biological communities of these once barren islands and may be the driving factor in these dramatic transformations. This study focuses on the northern *borealis* sub-species of common eider, but as the other sub-species of common eider have similar life histories it is likely that they also act as a bio-vector for nutrients throughout the distribution of the species as a whole. We therefore encourage more studies into this phenomenon to better understand the potential impacts of changing distribution and population dynamics of eiders in response to factors such as hunting, disease outbreaks, and climate change that are occurring in the circumpolar Arctic.

## Supplementary Information

Below is the link to the electronic supplementary material.

Supplementary file1 (DOCX 21 KB)
