# Supplementary material for: Seaduck engineers in the Arctic Archipelago: nesting eiders deliver marine nutrients and transform the chemistry of island soils, plants, and ponds
Source: Oecologia. 2021 Mar 6;195(4):1041–52. doi: 10.1007/s00442-021-04889-9 (PMC8052239; doi:10.1007/s00442-021-04889-9)
Supplement: Supplementary file 1 — Supplementary file1 (DOCX 21 KB) [file 442_2021_4889_MOESM1_ESM.docx]

**Appendix 1.** Physical characteristics of sampling sites. Sites were selected to minimize variation in size, distance from shore, and elevation, though this was complicated due to limits on the number of islands we were able to visit and natural variation.

| **High Eider** |  |  |  |  |  |
| --- | --- | --- | --- | --- | --- |
| *ID Code* | *Latitude* | *Longitude* | *Elevation (m)* | *Distance to Mainland (km)* | *Area (km2)* |
| A-044 | 64.2979 | -75.7871 | 10 | 7.57 | 0.08 |
| A-045 | 64.2856 | -75.7821 | 20 | 8.52 | 1.20 |
| A-054 | 64.3177 | -75.7547 | 10 | 4.96 | 0.10 |
| A-056 | 64.2832 | -75.7402 | 10 | 8.63 | 0.28 |
| A-083 | 64.3223 | -74.6669 | <10 | 7.41 | 0.04 |
| A-085 | 64.2923 | -74.6488 | 20 | 10.8 | 0.17 |
| A-108 | 64.3396 | -74.3759 | 10 | 9.09 | 0.15 |
| A-110 | 64.3186 | -74.3316 | 50 | 7.90 | 2.32 |
| A-112 | 64.2959 | -74.2679 | 20 | 7.82 | 0.13 |
| A-114 | 64.2683 | -74.189 | 20 | 7.03 | 0.39 |
| A-135 | 64.0622 | -73.5321 | 20 | 12.6 | 0.25 |
| A-136 | 64.0852 | -73.5123 | 20 | 9.81 | 0.12 |
| D-003 | 62.4312 | -78.1713 | 15 | 8.51 | 0.24 |
| D-004 | 62.4436 | -78.1506 | 15 | 9.24 | 0.19 |
| D-012 | 62.3836 | -78.185 | 15 | 5.28 | 0.11 |
| D-016 | 62.3222 | -78.1759 | 15 | 2.06 | 0.05 |
| D-022 | 62.4858 | -77.8767 | <10 | 5.39 | 0.07 |
|  |  |  |  |  |  |
| **Low Eider** |  |  |  |  |  |
| *ID Code* | *Latitude* | *Longitude* | *Elevation (m)* | *Distance to Mainland (km)* | *Area (km2)* |
| A-038 | 64.2476 | -76.2292 | <10 | 4.05 | 0.01 |
| A-043 | 64.2979 | -75.807 | <10 | 7.58 | 0.01 |
| A-143 | 64.1028 | -73.4571 | <10 | 7.22 | 0.04 |
| D-007 | 62.4454 | -78.1356 | <10 | 9.19 | 0.04 |
| D-019 | 62.3381 | -78.1583 | <10 | 2.04 | 0.03 |
|  |  |  |  |  |  |
| **Reference** |  |  |  |  |  |
| *ID Code* | *Latitude* | *Longitude* | *Elevation (m)* | *Distance to Mainland (km)* | *Area (km2)* |
| Cape Dorset (outside town) | 64.22356 | -76.523 | 64 | n/a | 507,451 |
| Ivujivik (outside town) | 62.39969 | -77.9098 | 66 | n/a | n/a |
| Baffin Reference #1 | 64.37259 | -74.5201 | 19 | n/a | 507,451 |
| Baffin Reference #2 | 64.37411 | -75.7543 | <10 | n/a | 507,451 |
| Baffin Reference #3 | 64.36808 | -76.1013 | <10 | n/a | 507,451 |
| DI-1 | 62.57202 | -77.6758 | 184 | 4.78 | 91.3 |
| DI-2 | 62.57108 | -77.6706 | 204 | 4.78 | 91.3 |
| DI-3 | 62.57063 | -77.6741 | 192 | 4.78 | 91.3 |
| DI-4 | 62.57301 | -77.6778 | 183 | 4.78 | 91.3 |
| DI-5 | 62.57848 | -77.6954 | 170 | 4.78 | 91.3 |
| DI-6 | 62.57384 | -77.7484 | 182 | 4.78 | 91.3 |
| DI-7 | 62.57649 | -77.7497 | 187 | 4.78 | 91.3 |
| DI-8 | 62.57891 | -77.8136 | 127 | 4.78 | 91.3 |
| DI-9 | 62.56803 | -77.8014 | 112 | 4.78 | 91.3 |
